# ACE-I/D Allele Modulates Improvements of Cardiorespiratory Function and Muscle Performance with Interval-Type Exercise

**DOI:** 10.3390/genes14051100

**Published:** 2023-05-17

**Authors:** Benedikt Gasser, David Niederseer, Walter O. Frey, Silvio Catuogno, Martin Flück

**Affiliations:** 1 Departement für Bewegung und Sport, Universität Basel, CH-4052 Basel, Switzerland; 2 Department of Cardiology, University Hospital Zurich, University of Zurich, CH-8008 Zurich, Switzerland; david.niederseer@usz.ch; 3 Swiss Olympic Medical Center, Balgrist University Hospital, CH-8008 Zurich, Switzerland; walter.frey@hin.ch (W.O.F.); silvio.catuogno@balgrist.ch (S.C.); 4 Laboratory for Muscle Plasticity, University of Zurich, Balgrist Campus, CH-8008 Zurich, Switzerland; 5 Department of Medicine, University of Fribourg, CH-1700 Fribourg, Switzerland

**Keywords:** aerobic combustion, eccentric training, softroboter, vasculariztation

## Abstract

***Background:*** The prominent insertion/deletion polymorphism in the gene for the major modulator of tissue perfusion, angiotensin-converting enzyme (*ACE-I/D*) is associated with variability in adjustments in cardiac and skeletal muscle performance with standard forms of endurance and strength type training. Here, we tested whether the *ACE-I/D* genotype would be associated with variability in the effects of interval-type training on peak and aerobic performance of peripheral muscle and cardio-vasculature and post-exercise recovery. ***Methods:*** Nine healthy subjects (39.0 ± 14.7 years of age; 64.6 ± 16.1 kg, 173.6 ± 9.9) completed eight weeks of interval training on a soft robotic device based on repeated sets of a pedaling exercise at a matched intensity relative to their peak aerobic power output. Prior to and post-training, peak anaerobic and aerobic power output was assessed, mechanical work and metabolic stress (oxygen saturation and hemoglobin concentrations of Musculus vastus lateralis (VAS) and Musculus gastrocnemius (GAS), blood lactate and factors setting cardiac output such as heart rate, systolic and diastolic blood pressure were monitored during ramp-incremental exercise and interval exercise with the calculation of areas under the curve (AUC), which were put in relation to the produced muscle work. Genotyping was performed based on I- and D-allele-specific polymerase chain reactions on genomic DNA from mucosal swaps. The significance of interaction effects between training and *ACE* I-allele on absolute and work-related values was assessed with repeated measures ANOVA. ***Results:*** Subjects delivered 87% more muscle work/power, 106% more cardiac output, and muscles experienced ~72% more of a deficit in oxygen saturation and a ~35% higher passage of total hemoglobin during single interval exercise after the eight weeks of training. Interval training affected aspects of skeletal muscle metabolism and performance, whose variability was associated with the *ACE* I-allele. This concerned the economically favorable alterations in the work-related AUC for the deficit of SmO_2_ in the VAS and GAS muscles during the ramp exercise for the I-allele carriers and opposing deteriorations in non-carriers. Conversely, oxygen saturation in the VAS and GAS at rest and during interval exercise was selectively improved after training for the non-carriers of the I-allele when the AUC of tHb per work during interval exercise deteriorated in the carriers. Training also improved aerobic peak power output by 4% in the carriers but not the non-carriers (*p* = 0.772) of the *ACE* I-allele while reducing negative peak power (−27.0%) to a lesser extent in the *ACE* I-allele carriers than the non-carriers. Variability in cardiac parameters (i.e., the AUC of heart rate and glucose during ramp exercise, was similar to the time to recovery of maximal tHb in both muscles after cessation of ramp exercise, only associated with the *ACE* I-allele but not training per se. Diastolic blood pressure and cardiac output during recovery from exhaustive ramp exercise demonstrated a trend for training-associated differences in association with the *ACE* I-allele. ***Discussion:*** The exercise-type dependent manifestation of antidromic adjustments in leg muscle perfusion and associated local aerobic metabolism between carriers and non-carriers of the *ACE* I-allele with the interval-training highlight that non-carriers of the I-allele do not present an essential handicap to improve perfusion-related aerobic muscle metabolism but that the manifestation of responsiveness depends on the produced work. ***Conclusions:*** The deployed interval-type of exercise produced *ACE* I-allele-related differences in the alterations of negative anaerobic performance and perfusion-related aerobic muscle metabolism, which manifestation is exercise specific. The training-invariant *ACE* I-allele-associated differences in heart rate and blood glucose concentration emphasize that the repeated impact of the interval stimulus, despite a near doubling of the initial metabolic load, was insufficient to overturn *ACE*-related genetic influences on cardiovascular function.

## 1. Introduction

Human performance is largely dependent on the anatomic design of the tissular pathways that connect energy metabolism to force production in the contracting skeletal muscle and adapt quantitatively and qualitatively to the repeated impacts of exercise [1]. It is thereby largely acknowledged that skeletal muscle and the associated cardiovascular system demonstrate a large degree of exercise-induced plasticity of tissue composition and function compared to other organs. Evidence has been provided concerning the association of a specific variant in certain genes (i.e., gene polymorphisms) with training-induced variability in exercise-induced alterations [2]. One factor contributing to interindividual differences in metabolic efficiency is vascularization, which yields in consequence to the prominent polymorphism of the angiotensin-converting enzyme (*ACE*) [3,4,5]. Thereby, the presence of a 287-base pair insertion (the I-allele) is associated with silencing the expression of the encoded *ACE* peptidase [6,7]. As a consequence of holding the I-allele, a reduced expression of *ACE* and consequently reduced processing of the precursor of the major myogenic regulator of smooth and cardiac muscle, the octapeptide angiotensin II, and the degradation of the vasodilative kinin peptides results [8,9]. Subjects that carry the *ACE* I-allele (so-called *ACE* deletion allele carriers) are typically characterized by a lowered peripheral blood flow during exercise due to a persistently higher peripheral vascular resistance [7,8]. Furthermore, the association of the *ACE* D-allele with left ventricular hypertrophy is well documented, and as a consequence, an elevated cardiac output is noted in non-I-allele carriers [10,11,12]. Additionally, and possibly interdependently, the influence of the *ACE*-I/D gene polymorphism on blood flow-dependent parameters interacts with the effects of physical activity.

In skeletal muscle, differences are described in terms of capillarization, mitochondrial volume density and the connected variability in lipid, glucose and amino acid metabolism [11,13], as well as the systemic cardiovascular system for maximal oxygen uptake, minute ventilation and arterial oxygen saturation [14,15]. The reported influences of the *ACE*-I/D gene polymorphism on human performance involve effects on metabolic efficiency and economy, including the better trainability of multiple aspects of the endurance-related trait of aerobic performance in the *ACE* I-allele carriers, especially those connecting oxygen delivery from the pulmonary level to contracting skeletal muscle [4,16]. Due to the respective association of the *ACE* I- and D-allele with training-induced variability of indicators of performance in endurance or power sports, the *ACE*-I/D gene polymorphism is considered to represent a biomarker of athletic prowess (reviewed by De Mello Costa et al., 2012) [17].

We recently reported that the interactions between the *ACE*-I/D genotype and aerobic fitness are associated with variability in the quantitative contribution of pulmonary, cardiovascular and muscle aspects of systemic aerobic metabolism to work production by contracting muscle [18]. Aerobic fitness and metabolic efficiency are explained by interacting influences of the *ACE*-I/D genotype on the oxygen transport system [18]. The implicated metabolic processes, which at the core probably include alterations in blood vessel perfusion [19] and the associated supply of oxygen to contracting skeletal muscle [18], are, however, only partly elucidated. Nevertheless, relevance is granted as the I-allele is reported to be enriched in subjects with an improved endurance trainability of muscle-based performance exceeding efforts beyond a few minutes [2,18].

Furthermore, it was previously reported that the *ACE*-I/D genotype may influence maximal whole-body aerobic metabolism but that this does not resolve in all instances due to the very small effect sizes for VO_2max_ and the contribution of VO_2_ and cardiac output to the produced power, irrespective of the genotype or if the interactions with the fitness state were considered [16,17,18]. Our observations imply that the contribution of the *ACE*-I/D genotype on the efficiency, and maybe economy, of aerobic metabolism and its fatigue resistance to fuel cycling muscle contractions is distinctly dependent on the level of physical activity and fitness state. Thereby, the homozygous D-allele carriers (i.e., non-carriers of the I-allele) were frequently shown to demonstrate the largest values for cardiac function, whereas I-allele carriers were found to show larger improvements in skeletal muscle’s aerobic capacity with training composed of continuous cycling exercise at constant workload [16,17,18]. It is, therefore, generally expected that the effects related to endurance performance would be larger in carriers than non-carriers of the I-allele, whereas cardiac function may show the inverse influence [16,17,18]. However, these aspects are not completely elucidated, especially regarding the varied types of exercise with different influences on perfusion-dependent aspects of skeletal muscle metabolism. Influences on muscle perfusion are, for instance, expected to result from a high load or lengthening of actively contracting skeletal muscle owing to the resulting compression or stretch of capillaries (reviewed in Durmic et al. [19,20,21]). This directly yields to the aim of this study to investigate the *ACE* I/D genotype-specific effects of interval exercise training, comprising a blend of eccentric and concentric contractions on short maximal muscle performance and endurance-related performance of peripheral muscle, cardio-respiration and aerobic muscle metabolism in healthy subjects. For the latter aspect, we quantified the selected endpoints that reflect the perfusion and oxygen metabolism of skeletal muscle at rest, during interval exercise and the exhaustive ramp exercise with near-infrared spectroscopy before and after training.

The expectation based on the literature was that the *ACE* I-allele carriers would demonstrate stronger improvements in aerobic muscle metabolism with training due to better perfusion, i.e., higher total hemoglobin content, during exercise and a faster and more complete replenishment of muscle oxygen saturation with the cessation of exercise after exhaustion. Conversely, we assumed that the cardiovascular aspects would be directly related to cardiac output and that the glucose uptake into skeletal muscle [10,11,22] would be worse in non-carriers of the *ACE* I-allele, but would eventually improve in the course of training owing to the repeated challenge to cardiometabolic and -vascular function during exercise. As a hypothesis with potential falsification, it shall be stated that no association between the *ACE* gene polymorphism and the training answer can be detected [23].

## 2. Material & Methods

***Subjects:*** Nine healthy subjects (39.0 ± 14.7 years of age; 64.6 ± 16.1 kg, 173.6 ± 9.9 cm) were recruited with a local advertisement via a flyer and word of mouth for a study into the “Effect of the *ACE* genotype on cardiovascular REHABilitation (*ACE*-REHAB)”. Two males (both an *ACE* I/D genotype) and seven females were analyzed. Physical healthiness was assessed with a health questionnaire. The absence of cardiovascular abnormalities was verified by a physician based on electrocardiographic measurement at rest and during the ramp-incremental exercise test with a 12-channel ECG (customized, Ottobrunn, Germany). The participants were advised to perform an eight-week training program appearing fit and well-nourished at the training and testing session. The study was in line with the Declaration of Helsinki and its later amendments and ethics approval was obtained by the ethics committee of the Canton of Zurich (Basec nr: 2016-02060) and registered on the clinical trials register (https://clinicaltrials.gov/ct2/show/NCT02845063 (accessed on 18 April 2023)).

***Design:*** Participants completed eight weeks of interval training on a soft robot. Before and after training, test sessions on the soft robot and standardized ramp-incremental exercise on a cycle ergometer were conducted to assess anaerobic strength (based on positive, negative and reactive peak power and corresponding rates of force development) and systemic and local aerobic capacity (based on peak power output and maximal oxygen uptake (VO2max), and muscle oxygen saturation and total hemoglobin concentration). The subjects were familiarized with the interval exercise before the first full session of the interval exercise. Each exercise session was supervised by a qualified sports scientist. During the ramp-incremental exercise, the first and last interval exercise session, and the first 8 min of the respective recovery after cessation of ramp exercise, measurements of cardio-respiration, muscle oxygenation and blood metabolites were conducted. The rate of perceived exertion was assessed each second minute with the Borg scale [24]. Genotyping was subsequently performed.

***Sessions of interval exercise:*** The interval training was based on repeated sets of pedaling exercises with a mix of concentric (positive work) and eccentric (negative work) contractions on a soft robot with modifications as described [25]. Each interval consisted of 1 min of work being interspersed by 1 min of rest with the legs resting on the pedal. Subjects conducted three sessions of interval exercise per week on alternate days at an initial target workload corresponding to 65% of aerobic peak power output as determined in the ramp exercise. The developed power, work produced per exercise session and share of positive versus negative work was monitored and supervised by a qualified sports scientist (see Table S1). At the end of each two-week phase of training, the number of sets and/or target power of interval exercise was progressively increased, respectively, every 2 weeks in case the previously targeted workload could be performed as advised. The percentage contribution of positive vs. negative work produced by either leg during a session of interval exercise did not differ both before and after the training intervention (*p* > 0.4, Table S1).

***Soft robotic device:*** The Allegro Medical Softroboter (Dynamic Devices AG/Zürich/Switzerland) is a training and rehabilitation gear with the function of a dynamic leg press. It allows for concentric and eccentric muscle work [26]. For positive and negative muscle work, reliance is mainly on the Musculus gluteus maximus, Musculus quadriceps femoris, Musculus biceps femoris, Musculus semimembranosus, and Musculus semitendinosus. For training, both legs were separately coupled during the whole exercise session, and the legs had to move back in a parallel, standardized and calibrated position where the distance to perform muscle work was around 0.5 m. Visual feedback was provided during exercise via a screen to allow the subject to control leg moment and dose the developed power. The maximal values of power and rate of force development during positive, negative and reactive single-leg extensions were assessed with specific tests on the soft robotic device as described [25].

***Ramp-incremental exercise to exhaustion:*** Before the eight-week training session and after a classic cardiopulmonary exercise, a cycle was tested. The test protocol was conducted in an air-conditioned laboratory at a standardized temperature of 20 °C according to a modified version of a published protocol [27]. In brief, the test started with three minutes of rest, when the subjects sat still on the cycle ergometer while maintaining a normal breathing pattern without warming up; subsequently, subjects started pedaling at an initial power (75 W for women and 100 W for men). Target power was increased every 20 s (18 W∙min^−1^ for women and 30 W∙min^−1^ for men). The subjects were asked to keep a constant self-chosen pedal cadence between 70 and 100 rpm throughout the test optimally. The test was stopped when the subjects experienced volitional exhaustion and/or were not able to maintain the target pedal cadence. Subsequently, recordings continued for a period of eight minutes when subjects rested in a seated position on the cycle ergometer.

***Measurements of cardio-respiration:*** Respiratory parameters (RF, VCO_2_, VO_2_), heart rate, and arterial oxygen saturation (SpO_2_) were continuously monitored with validated protocols and equipment, i.e., Metalyzer 3B-R2 device (Cortex, Leipzig, Germany), pulse belt (Suunto, Vantaa, Finland) and a pulsoxymeter (Sonomed GMBH, Volketswil, Zürich, Switzerland). Measurements of systolic and diastolic blood pressure were manually conducted every two minutes (SunTech Medical, Morrisville, NC, USA) and used to estimate cardiac output based on the assessed parameters according to Liljestrand and Zander [13] with a constant correction factor of 0.302 as estimated from Innocor measurements of the effective cardiac output during comparable exercise [26].

***Measurements of muscle oxygenation:*** During the entire duration of the ramp-incremental exercise and first and last interval exercise power output, SmO_2_ and THb in a knee extensor muscle (VAS) and a plantar flexor muscle (GAS) were monitored at 2 Hz based on near-infrared spectroscopy to estimate mitochondrial respiration [28] and perfusion, respectively [24], with a portable muscle oxygen monitor (Moxy, Fortiori Design LLC, Hutchinson, MN, USA), which was also used to measure hemoglobin non-invasively as previously described by us and others [28,29,30,31,32]. The device uses four different light sources covering wavelengths in the range of 630 to 850 nm and a modified Beer–Lambert law to perform measurements of SmO_2_. The sensor was placed on the lower third of VAS in the middle of the muscle belly on the left leg of the subjects. Prior to the placement, the skin site was shaved using a disposable razor (Gallant, Dynarex, Orangeburg, NC, USA) and cleaned with an alcohol swab (Webcol™, Covidien™, Dublin, Ireland). The sensor was attached using the recommended tape (Moxy Adhesive Attachments, Fortiori Design LLC, Spicer, MN, USA). To protect the NIRS device from ambient light, it was covered with an adhesive non-woven fabric (Hypafix^®^, BSNmedical, Hamburg, Germany).

***Measurement of blood glucose and lactate:*** Two 20 µL aliquots of capillary blood were drawn every two minutes during interval exercise, ramp exercise, and the subsequent first 8 min after ramp exercise from the ear lobe and subjected to the quantification of glucose using the OneTouch^®^ Vita™ Blood Glucose Monitoring System (Lifescan, Milpitas, CA, USA) or lactate using a Biosen C-line analyzer (EKF Diagnostic GmbH, Barleben, Germany).

***Genotyping:*** The typing of the *ACE*-I/D gene polymorphism was carried out with modifications of the described polymerase chain reaction protocol [32]. In brief, genomic material was extracted from fresh buccal swabs by applying a commercially available protocol that comprised the degradation of ribonucleic acid and proteins based on RNAse and proteinase K and a last step of filtration with a QIAamp Mini spin column (QIAamp^®^ DNA Mini Kit, Qiagen, Hilden, Germany). Two microliters of the resulting eluate (150 µL in total) were subjected to a polymerase chain reaction in 48-well plates with a specific combination of primer sets followed by high-resolution melt analysis with a real-time PCR system (Eco™, illumina^®^, San Diego, CA, USA) as described [8]. The primer mix for the detection of the I-allele specific 66 bp amplicon contained the primer *ACE*2 (5′-tgggattacaggcgtgatacag-3′) and the primer *ACE*3 (5′atttcagagctggaataaaatt-3′). The primer mix for the detection of the D-allele specific 83 bp amplicon contained primers *ACE*1 (5′-catcctttctcccatttctc-3′) and *ACE*3 (5′atttcagagctggaataaaatt-3′). The applied reaction conditions included a denaturation step for 10 min at 95 °C and 40 cycles of amplification comprising a denaturation at 95 °C for 15 s, and annealing/extension at 55 °C for 1 min. The products were subsequently analyzed based on the melting curve between 60 °C and 95 °C. Genotype analysis was done using genetic variation analysis software (EcoStudy Version 5.0, Illumina^®^, San Diego, CA, USA).

***Data handling:*** Data from the various devices were imported into Microsoft Excel (Microsoft Inc., Redmond, WA, USA) and curated and used to extract the respective values and estimate the respective total areas under the curve (AUC) by the trapezoidal rule, essentially as described [26,33]. The calculations concerned the estimates for the total skeletal muscle work and the accretion of values for the indices of cardio-respiratory work (cardiac output, heart rate, systolic/diastolic blood pressure, muscle metabolism (SmO_2_ deficit, and tHb concentration) and concentrations of blood glucose and lactate. The estimated ‘absolute’ values characterizing the exercise of a given subject were also related to the total muscle work performed in the respective exercise to estimate muscle-specific work capacity.

***Statistical analyses:*** The mean, SD, and SE were calculated pre- versus post- for the exercise test. Pre/post training differences in the estimated parameters and associations with the *ACE*-I/D genotype were calculated separately for each parameter with repeated-measures ANOVA for the repeated factor pre/post training and the factor *ACE* I-allele (yes/no). The post-hoc differences were calculated with a test for the least significant difference. The calculations were made with Microsoft Excel (Microsoft Inc., Redmond, WA, USA) and SPSS (Armonk, New York, NY, USA).

## 3. Results

**Figure 1:**
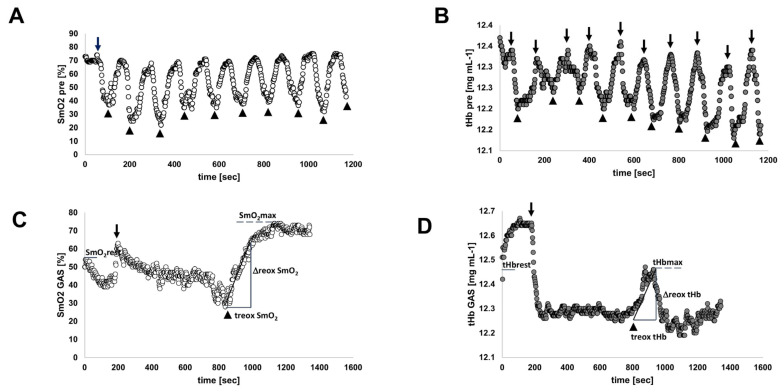
**Perfusion-related muscle metabolism during interval and ramp exercise.** Scatter plot of examples of oxygen saturation (**A**) and tHb concentration (**B**) in GAS muscle during interval exercise. (**C**,**D**) Oxygen saturation (**C**) and tHb concentration (**D**) in one subject during the ramp exercise to exhaustion before training. Pedaling started at 200 s and stopped near 834 s. The extracted parameters are exemplarily indicated in (**C**,**D**). Arrows and arrowheads denote the start and end, respectively, of pedaling.

ACE-I/D genotype-associated differences in muscle strength, skeletal muscle, and cardiac metabolism before training—Table 1, Table 2 and Table 3 provide an overview of the performance and metabolic characteristics of the subjects as assessed at baseline before interval training during anaerobic strength tests and exhaustive ramp-incremental ergometer exercise. The indices of aerobic and anaerobic strength did not differ between carriers and non-carriers of the *ACE* I-allele, although a trend for better performance was indicated for *ACE* I-allele carriers (Table 1). Equally, the indices of cardio-respiratory work and muscle metabolism during the ramp exercise did not vary in association with the *ACE* I-allele (Table 2 and Table 3). Figure 1 provides an example of perfusion-related muscle metabolism during ramp exercise.

Characteristics of the interval-type training stimulus—The subjects started training with sessions of pedaling interval exercises at a constant intensity corresponding to 65% relative to maximal aerobic power output (PPO). The oxygen saturation and tHb concentration in the two studied leg muscles, GAS and VAS, were typically lowered during the 1 min intervals of pedaling and recovered during each subsequent period of rest (Figure 1).

**Figure 2:**
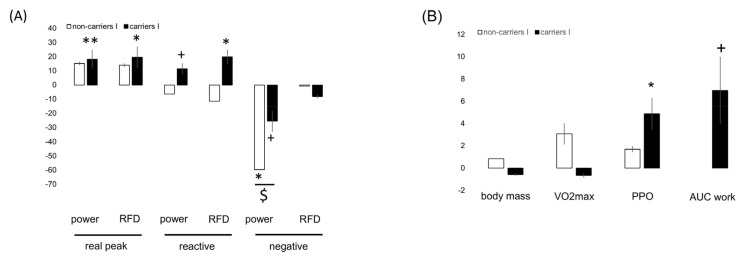

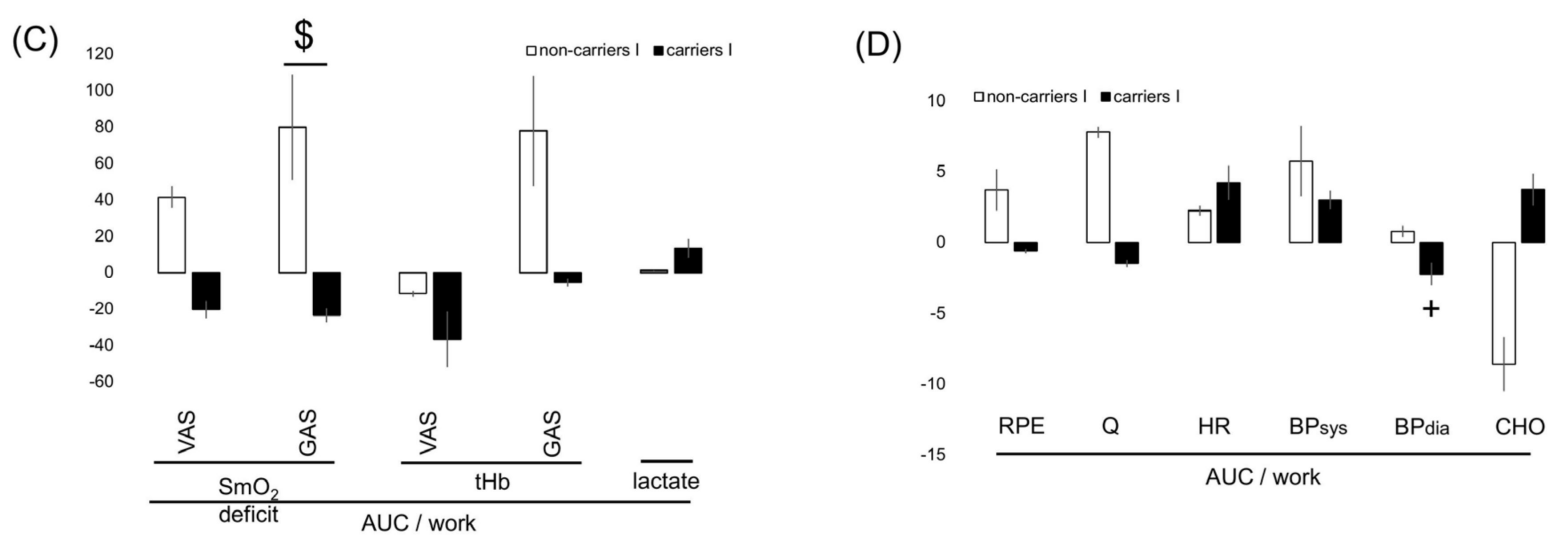
**Interval training-induced effects on physiological performance characteristics between genotypes.** Bar graph of the mean ± SD of interval training-induced effects (i.e., after vs. before 8 weeks of interval-type training) between *ACE*-I/D genotypes for parameters characterizing muscle performance (**A**), aerobic capacity (**B**), aerobic muscle metabolism (**C**), and cardiovascular function (**D**) during incremental exercise. Note the antidromic influence of interval type training between the *ACE* I/D genotypes for I-allele carriers vs. no I-allele carriers for the situation post- versus pre-. +, *p* < 0.10; *, ** *p* < 0.05 and 0.01. $, 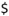 *p* < 0.05. (for post- vs. pre-, meaning pre- versus post-training intervention).

Interval exercise produced a substantial percentage reduction in the average oxygen saturation in VAS (−46.2 ± 29.4%) and GAS muscle (−57.0 ± −40.2%) respective to the situation at rest. The concentration of total hemoglobin was moderately affected during interval exercise in VAS (−0.5%, *p* = 0.12) and GAS muscle (−0.9%, *p* = 0.14), whereby the average tHb concentration during exercise amounted to 12.2 ± 0.5 mg mL^−1^ and 12.4 ± 0.6 mg mL^−1^, respectively (Figure 2).

The targeted power to be delivered during sets of interval exercises was progressively increased during the 8 weeks of interval training (see Supplementary Table S1). The percentage contribution of positive work to the total work delivered during interval exercise did not differ between the last and first interval exercise sessions (*p* = 0.625). At the end of the training, the ‘absolute’, and PPO-related target power was increased by 87%. In consequence, also the metabolic demand for cardiac and skeletal muscle during interval exercise was increased with training (Supplementary Table S2). Thereby, the accrued values, i.e., AUCs, for indices of metabolic stress during interval exercise were increased by 106% for cardiac output, 35% for tHb in VAS and 37% in GAS, and 52% for blood glucose concentration (during interval exercise, after training, whereas work-related metabolic demand (i.e., AUC per work) was less affected, yet the SmO_2_ deficit per work during interval exercise was reduced both in VAS (−12.5%) and GAS (−15.2%) muscle after interval training (Supplementary Table S3).

Effects of interval training on performance and metabolism and its association with the ACE-I/D genotype—An *ACE*-I/D-genotype-associated difference was noted for physiological parameters of aerobic metabolism. This concerned an 83% higher cardiac output and 29% higher heart rate in non-carriers of the *ACE* I-allele at rest, when the SmO_2_ in GAS muscle was 25% higher in I-allele carriers. (Table 4).

**Figure 3:**
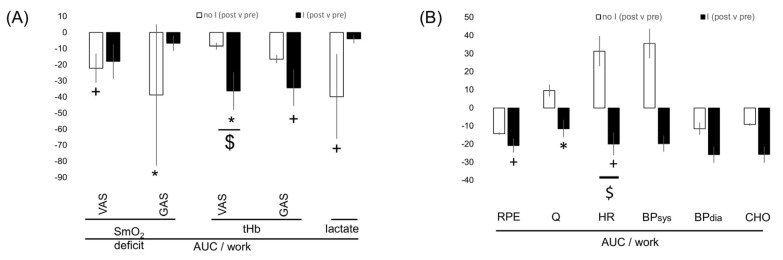
***ACE* genotype difference for training-induced effects on aerobic muscle metabolism during interval exercise.** Bar graph of the mean ± SD of interval training-induced effects per *ACE*-I/D genotype (i.e., after vs. before 8 weeks of interval-type training) for parameters characterizing perfusion-related aerobic muscle function (**A**) and cardiovascular function (**B**) during interval exercise. Note the tendency for *ACE* I/D genotypes related to differences in training effects on the AUC of SmO_2_ deficit and aspects of cardiovascular functioning during exercise. +, *p* < 0.10; *, *p* < 0.05. $, 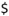 (for post- vs. pre-, meaning pre- versus post-training intervention).

**Figure 4:**
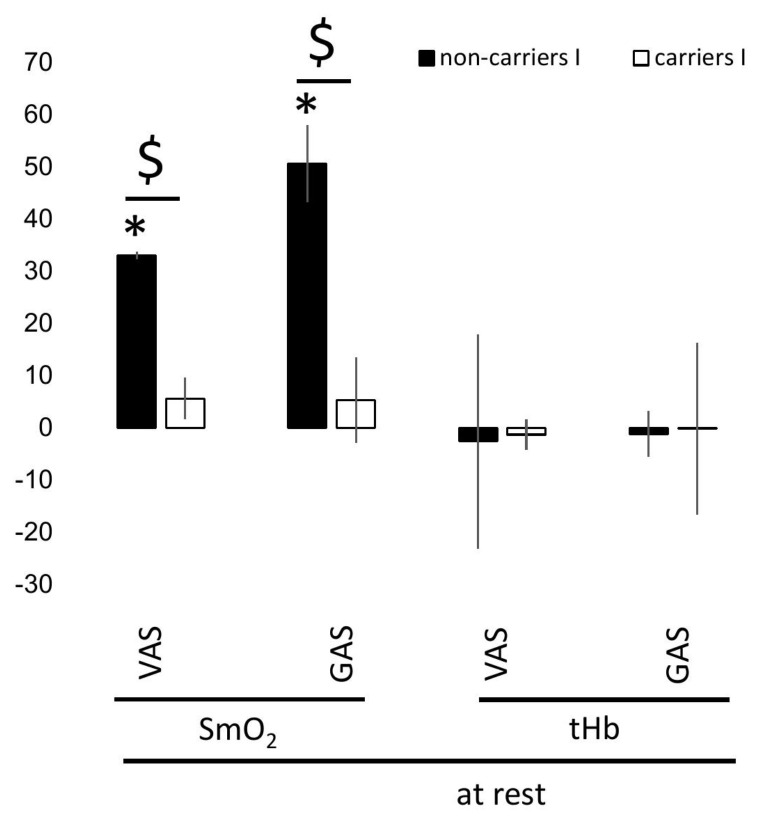
**Interval training-induced effects on cardiovascular function between genotypes**. Bar graph of the mean ± SD of interval training-induced effects per *ACE*-I/D genotype (i.e., after vs. before 8 weeks of interval-type training) for parameters characterizing aerobic muscle metabolism at rest before ramp exercise. *, *p* < 0.05. 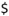, *p* < 0.05 I vs. no I (for post- vs. pre-, meaning pre- versus post-training intervention).

**Figure 5:**
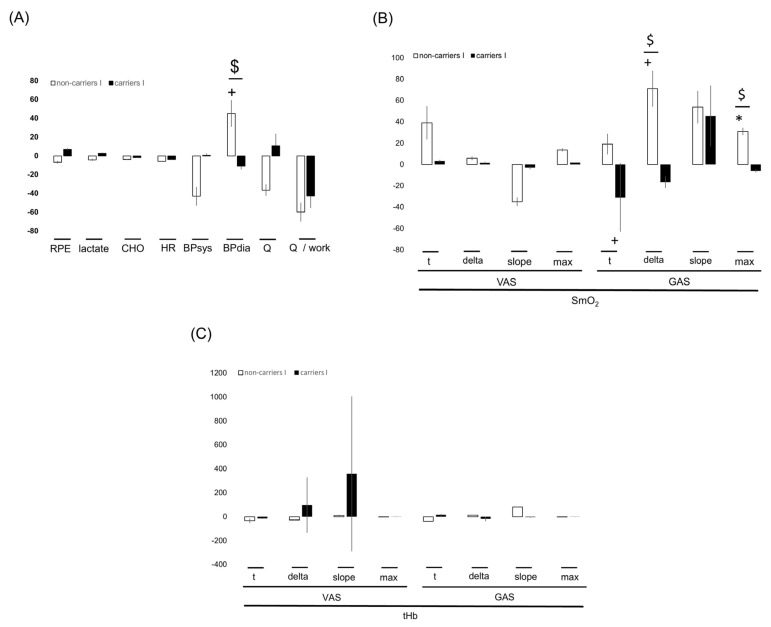
***ACE* genotype difference for interval training-induced effects on recovery of cardiac function post-exercise**. Bar graph of the mean ± SD of interval training-induced effects per *ACE*-I/D genotype (i.e., after vs. before 8 weeks of interval-type training) for parameters characterizing recovery of cardiac function (**A**) and muscle metabolism (i.e., SmO_2_, (**B**); tHb, (**C**)) after incremental ramp exercise. Note the influence of interval type training between the *ACE* I/D genotypes for diastolic blood pressure, which runs antidromic to the influence on systolic blood pressure and the antidromic influence of interval type training between the *ACE* I/D genotypes for muscle oxygenation in GAS. +, *p* < 0.10; *, *p* < 0.05. $, 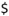 *p* < 0.05 I vs. no I (for post- vs. pre-, meaning pre- versus post-training intervention).

Supplementary Tables S4–S6 and Figure 2, Figure 3, Figure 4 and Figure 5 summarize the significance and magnitude of interval training effects on skeletal muscle and cardiovascular performance and perfusion-related metabolism during ramp exercise and interval exercise.

Interval-type training increased positive peak power (+21.8%) and reduced negative peak power (−27.0%; Table S4). Aerobic peak power output was (overall) not affected by the training (*p* = 0.189), but it was 4% improved in the I-allele carriers with training (Figure 4, Supplementary Table S4). Similarly, the work-related AUC for the deficit in SmO_2_ during incremental ramp exercise in both the VAS and GAS muscles was affected by training in association with the *ACE* I-allele (Supplementary Table S5; Figure 4).

Similarly, the work-related AUC of the deficit in SmO_2_ in VAS and GAS muscle during interval exercise were affected by the interaction between training and the *ACE* I-allele (Supplementary Table S6). Lactate per work during interval exercise demonstrated a trend for an interaction between the effect of training and the *ACE* I-allele, which was localized to differences between I-allele carriers and non-carriers before training (Figure 4).

From the work-related values for parameters of cardiovascular function during and/or interval ramp exercise, the AUC of heart rate per work during interval exercise tended to be affected by the interaction of training and *ACE* I-allele (Supplementary Tables S5 and S6, Figure 2).

ACE I-allele-associated training effects on metabolism at rest and during recovery from exercise—Supplementary Table S7 summarizes the significance and magnitude of interval training effects on the response of cardiovascular and perfusion-related skeletal muscle metabolism at rest. Training elevated the oxygen saturation in VAS (+14.2%) and GAS (+15.0%) muscle at rest, in association with the *ACE* I-allele, due to reactions in the non-carriers of the I-allele. tHb concentration at rest was moderately reduced after training in VAS (−1.6%) and independent of the *ACE*-I/D genotype (Supplementary Table S7).

Table S7 and Figure 5 summarize the significance and magnitude of interval training effects on the response of cardiovascular and perfusion-related skeletal muscle metabolism during recovery from voluntary ramp exercise to exhaustion. Parameters characterizing recovery of cardiac and muscle metabolism from ramp incremental exercise were not generally affected by training (Supplementary Table S7). Diastolic blood pressure and cardiac output during the first 8 min of recovery after ramp exercise demonstrated a trend for an I-allele-dependent effect of training. For diastolic blood pressure, this was explained by a selective training-induced increase in the values for this hemodynamic parameter in non-carriers of the *ACE* I-allele. Further on, a post-hoc difference was identified on the overshoot of SmO2 in GAS muscle during recovery from exhaustive ramp exercise, for which a trend of an interaction effect between training and *ACE* I-allele existed (Supplementary Table S7; Figure 4).

Variability in metabolic characteristics was mainly associated with the *ACE-I*-allele—Variability in the AUC of blood glucose concentration per work and the AUC of heart rate per work during exhaustive ramp exercise and subsequent recovery demonstrated an association with the *ACE* I-allele, which did not interact with the effect of training (Supplementary Table S5). However, when training-induced fold changes differed between carriers and non-carriers of the *ACE* I-allele. For both, blood glucose concentration and heart rate, this was reflected in substantially lower values in the *ACE* I-allele carriers than non-carriers (i.e., −30–50%; Figures S1–S3). Similarly, the work-related AUC of cardiac output and systolic blood pressure during ramp incremental exercise differed between carriers and non-carriers of the *ACE* I-allele after training (Supplementary Table S5; Figure S1).

Furthermore, the time to the maximal increase in tHb (i.e., treox tHb) in the GAS and VAS muscles during recovery from ramp exercise, was associated with the *ACE* I-allele, in trends (*p* = 0.130, 0.054) for the interactions with training. At the post-hoc level, this localized to higher values for treox tHb in the GAS in non-carriers compared to carriers of the *ACE* I-allele prior to training, which faded after training. The opposite trend was observed for the VAS muscle (Figure S1).

## 4. Discussion

Angiotensin-converting enzyme is a major regulator of vascular tone, and the insertion/deletion polymorphism within the encoding gene (i.e., *ACE*-I/D) has been found to be prominently associated with the variability in the blood pressure and cardiac growth response to repeated increases in hemodynamic stress [8,33,34,35]. This relays to inter-individual differences in training-induced adaptations of human performance that rely on cardiovascular function, such as the provision and combustion of blood-borne substrates for muscle’s energy metabolism. Recently, we reported that aerobic fitness-related variability in peripheral muscle oxygenation during exhaustive exercise is associated with the *ACE* I-allele [18]. Herein, we explore and identify that the adaptations of perfusion-related aspects of aerobic muscle metabolism (i.e., muscle oxygen saturation and tHb concentration), and negative peak power with interval-type training differ distinctly between the carriers and non-carriers of the *ACE* I-allele. In contrast, the *ACE* I-allele-associated aspects of cardiovascular function, i.e., blood glucose concentration and heart rate during exercise, were not essentially affected by the deployed eight weeks of training on a soft robot. Interval exercise of pedaling exercise provides a potentially more complete stimulus than pedaling exercise with a constant workload to enhance both anaerobic and aerobic performance and metabolism [8,33,34,35]. In this respect, it is worth noting that we identify a number of functionally important consequences of the deployed interval exercise. This includes improved anaerobic peak positive power and aspects of aerobic muscle metabolism, such as muscle oxygen saturation and tHb concentration at rest, the latter of which is, to the best of our knowledge, for the first time reported. Conversely, negative peak power, which capacity may negatively impact muscle perfusion due to stretch-induced constriction of muscle blood vessels during lengthening contraction was lowered after interval-type training and was pronounced for non-carriers of the ACE I-allele [36,37]. Overall, these observations point to a reduction in obstructive influences on muscle perfusion with interval training.

Interestingly and importantly, training effects on muscle oxygen saturation in the two studied leg muscles during ramp and interval exercise only resolved when the interaction with the *ACE* I-allele was considered. Thereby, a robust decrease resolved for the work-related AUC of the deficit of muscle oxygen saturation during ramp exercise in the *ACE* I-allele carriers, which also demonstrated a moderate (i.e., 4%) enhancement of aerobic power output. Importantly, the *ACE* I-allele-associated differences in training-induced adjustments of SmO_2_ per work did not relate to the differences before training, emphasizing that the observation is a bona fide example of the interaction between the studied genetic and conditional factors, i.e., the concept of the reciprocal influence of nature and nurture on a physiological trait [38].

The observations on muscle oxygen saturation per work indicate improved metabolic efficiency in the *ACE* I-allele carriers during a primarily aerobic exercise with interval training. Muscle oxygen saturation reflects the muscle’s mitochondrial capacity [28], indicating a reduced ‘respiration per muscle work at the selected target power in the *ACE* I-allele carriers during exhaustive ramp exercise. The observations imply that suggested *ACE* I-allele-related improvements of energy supply to contracting skeletal muscle with interval training do not comprise an improved local aerobic combustion of imported substrates. Indifferent levels of blood lactate concentration and VO_2max_ during ramp exercise with training indicate that the possible implication biochemical process includes improved anaerobic combustion of organic substrates in the studied leg muscles. In this respect, it is worth considering that the deployed interval exercise comprised a considerable percentage (i.e., 46%) of negative work, which has been found to possibly exert a negative influence on mitochondrial volume density [39] for which we have reported *ACE* I-allele-associated effects of physical training [40].

The closer inspection revealed that training effects on the work-related deficit in oxygen saturation differed between ramp and interval exercise. Thereby, for interval exercise, it was the non-carriers of the *ACE* I-allele, and not the I-allele carriers as seen during ramp exercise, that demonstrated a reduced work-related oxygen deficit after training. Correspondingly, *ACE* I-allele carriers demonstrated a reduced work-related AUC for total hemoglobin concentration during interval exercise. Interestingly, in the situation of interval exercise alone, a trend for a lowered accumulation of blood lactate concentration per work was noted in non-carriers of the *ACE* I-allele, pointing to the metabolic relevance of tHb and SmO_2_ related *ACE*-I/D genotype effects with training under constraining interval type of exercise.

tHb concentration (*ceteribus paribus* unchanged hematocrit) is a proxy for capillary blood content in the assessed voxel and, correspondingly, muscle perfusion [18,28]. Under the given condition, we interpret the observations by training-induced improvements in the resistance of blood vessels to withstand compressive forces during muscle contraction [20]. This would be consistent with relatively better protection of *ACE*-DD genotypes from the occlusive influence of interval exercise on the soft robot compared to I-allele carriers. It is interesting that types of exercise training with large eccentric components, such as downhill skiing, have been found to increase the capillary-to-fiber ratio in association with the *ACE* I/D genotype [19,41].

The interpretation of *ACE* I-allele-related effects on muscle perfusion during interval exercise has to be seen in relation to the trend for a higher absolute target power that the I-allele carriers had to deliver during interval exercise since the exercise intensity was matched to the constant proportion of peak aerobic power output. This notion is supported by the observation that the VAS and GAS muscles were subject to substantially higher fold differences in absolute (i.e., VAS/GAS: 13/24-fold) and work-related SmO_2_ deficits 175/287-fold) and absolute (360/467-fold) and work-related (5297/7204) cumulated tHb concentrations peer work during interval than ramp exercise. This difference is the probable result of the specific setup where both legs of the subjects had to constantly and independently apply pressure against the pedals to comply with the target requirements on the share between positive and negative power [42]. In this respect, it is of interest that the differences in metabolic stress between interval and ramp exercise were larger for the GAS compared to the VAS muscle in terms of the strain of the oxygen deficit (~+70%) and tHb concentration (+~35%). This may possibly explain the higher significance of training effects in perfusion-related variables during interval exercise in GAS compared to VAS.

We also observed *ACE*-I-allele-associated differences in training-induced alterations of the AUC of diastolic blood pressure and GAS muscle reoxygenation during the 8 min after stopping the exhaustive ramp exercise. This indicates that the recovery of blood flow-related cardiac and skeletal muscle aspects of metabolism from exhaustive leg exercise considerably and differently improved between the *ACE*-I/D genotypes with the deployed paradigm of interval training. Conversely, our findings on differences in the AUC of blood glucose concentration and AUC of heart rate (per delivered muscle work) between carriers and non-carriers of the *ACE* I-allele during ramp exercise and subsequent recovery confirm the reported association of variability in aspects of cardiovascular functioning with the *ACE*-I/D genotype [3,32]. We have recently reported that the biochemical processes involved in glucose uptake into muscle fibers are reduced in the *ACE*-DD genotypes in correspondence with elevated levels of angiotensin 2 [22]. This suggests that the training resistance of blood glucose concentration may be related to the persistent influence of different levels of angiotensin signaling in the capillary bed that supplies muscle fibers.

The unaffected cardiovascular parameters emphasize that the central (i.e., cardiac) effects of the deployed type of 8 weeks of repeated interval exercise were moderate compared to the adaptation at the skeletal muscle level. High-intensity interval training of a comparable duration of sets (i.e., 60 s) and number (i.e., 8–10) of sets of exercise but at a higher intensity relative to peak power output (i.e., 90–110% vs. 65%) has been found to increase cardiac output to a greater and significant extent as seen in our investigation [42]. In fact, the intensity and volumes were designed as a rehabilitative treatment for cardiac patients with central limitations [42,43]. In this respect, we indicate that borderline significant trends for training and genotype interactions for the work-related AUC of heart rate during interval exercise were noted in the *ACE* I-allele carriers after training. Our observations suggest that the stress on cardiac metabolism by the deployed exercise paradigm was too low to produce major cardiac adaptations.

Our findings provide useful knowledge for refining applications in the testing and training of sportsmen. The measurements of a muscle’s oxygen saturation and hemoglobin concentration during a workout are increasingly popular for monitoring training responses, especially as they provide information on the specific efficiency by which an exercising muscle is supplied with oxygen and uses oxygen during a workout [44]. Importantly, differences in the impacting metabolic and metabolic stress on skeletal muscle during a workout stand in proportion to the adaptations in the metabolic makeup of skeletal muscle with training (reviewed in Hoppeler et al., 2011 [45]). The *ACE*-I/D genotype-related individual differences in the transients of SmO_2_ and tHb discussed here indicate that information gleaned by genetic and NIRS measurements on *ACE*-I/D associated aerobic metabolism in skeletal muscle, and cardiovascular reactions, can be useful to tailor exercise interventions to provide a sufficient metabolic/hemodynamic stimulus to produce adaptive reactions of the cardiovascular and muscle metabolism that may overcome muscle perfusion-related bottlenecks in endurance performance. For instance, joint measurements on the specific *ACE*-I/D genotype, SmO_2_ and muscle tHb, provide valuable hints on how an exercise stimulus may be altered in dependence on whether a subject carries the *ACE* I or D-allele to increase or lower the degree of a contraction-induced stimulus of local hypoxia to produce an equal stimulus for the ensuing adaptations, such as improvements in local aerobic capacity due to mitochondrial biogenesis or angiogenesis [6,19]. Similarly, information on the specific *ACE*-I/D genotype may be useful to predict improvements in the capacity to recover muscle oxygen metabolism and diastolic blood pressure from exhaustive exercise. The present novel observations motivate future investigations to address the quantitative relationship between *ACE*-I/D genotype, training status and exercise intensity for adaptations in muscle oxygen saturation and perfusion with systematic forms of (interval-type) training.

Limitations: We highlight that certain experimental influences may have contributed to our interpretation of the results. Firstly. Because subjects demonstrated differences in muscle power before training, we chose to relate values for the associated metabolic expenditure (i.e., AUC for parameters being associated with cardiac output and muscle perfusion/oxygen saturation) to the target workload during the tasks of interval and incremental ramp exercises to compare training and *ACE* I-allele-related inter-individual differences.

## 5. Conclusions

Interval-type training affected the aerobic and anaerobic performances of skeletal muscle, which except for negative peak power, did not differ in association with the *ACE* I-allele. Concurrently, the *ACE* I-allele-specific improvements of skeletal muscle’s (aerobic) metabolic economy and perfusion during exhaustive ramp and/or interval exercise revealed after interval type training (based on the accrued muscle oxygen deficit and tHb concentration), and this was exercise-type dependent. These effects were more pronounced for the GAS compared to VAS muscle and also manifested in an improved reoxygenation in non-carriers of the *ACE* I-allele post-exercise in correspondence with training-induced improvements of muscle perfusion (i.e., elevated tHb concentration). Aspects of cardiovascular functioning, such as blood glucose concentration and heart rate during ramp exercise (per delivered muscle work) and subsequent recovery, were robustly elevated in non-carriers of the *ACE* I-allele [42] and only diastolic blood pressure demonstrated a trend of interaction with training effect identified during recovery from exhaustive exercise. Our findings further support the notion that the *ACE*-I/D genotype contributes to inter-individual influences of interval training on skeletal muscle’s aerobic metabolic efficiency via a mechanism that involves altered muscle perfusion.

## Figures and Tables

**Table 1:** Performance characteristics of the subjects before training. Mean ± SE of values over all (*n* = 9), carriers (*n* = 7) and non-carriers (*n* = 2) of the *ACE* I-allele and *p*-value of the effect of the I-allele (ANOVA) on characteristics of performance.

	*All*	*Non-Carriers*	*I-Allele Carriers*	*p-Value*
body mass [kg m^−2^]	65.8 ± 8.8	59.0 ± 0.0	67.8 ± 11.3	0.327
VO_2max_ [mL min^−1^ kg^−1^]	43.1 ± 12.8	32.5 ± 9.7	46.1 ± 13.7	0.239
PPO [Watt]	253.0 ± 64.5	177.0 ± 25.5	274.7 ± 75.7	0.129
AUC work [kJ]	86.4 ± 30.7	47.5 ± 15.5	97.5 ± 35.0	0.140
positive peak power [W]	779.7 ± 316.8	624.5 ± 79.3	824.1 ± 384.7	0.508
positive peak RFD [N s^−1^]	4425.4 ± 2069.6	3858.9 ± 455.7	4587.2 ± 2530.7	0.710
reactive peak power [W]	923.5 ± 342.9	763.7 ± 132.7	969.1 ± 403.0	0.518
reactive peak RFD [N s^−1^]	4948.4 ± 1904.8	4286.2 ± 723.6	5137.6 ± 2242.3	0.628
negative peak power [W]	382.3 ± 156.2	416.1 ± 49.2	372.6 ± 186.8	0.764
negative peak RFD [N s^−1^]	4126.9 ± 949.9	3440.6 ± 129.5	4323.0 ± 1184.3	0.349

**Table 2:** Cardiovascular function of the subjects before training. Mean ± SE of values over all (*n* = 9), carriers (*n* = 7) and non-carriers (*n* = 2) of the *ACE* I-allele and *p*-value of the effect of the I-allele (repeated-measures ANOVA for the training and *ACE*-I-allele factors) on characteristics of performance during ramp exercise.

	*All*	*Non-Carriers*	*I-Allele Carriers*	*p-Value*
peak heart rate [beats min^−1^]	176.0 ± 10.5	175.5 ± 21.9	176.1 ± 7.3	0.189
AUC cardiac output [L]	1791.5 ± 622.6	1331.15 ± 578.22	1922.99 ± 635.27	0.427
AUC heart rate [1000 × bpm s]	85.7 ± 19.8	73.3 ±20.8	89.3 ± 19.5	0.982
AUC systolic BP [mmHg s × 1000]	104.8 ± 19.4	84.2 ± 2.5	110.7 ± 24.2	0.779
AUC diastolic BP [mmHg s × 1000]	51.0 ± 7.5	45.2 ± 3.5	52.7 ± 8.7	0.907
AUC blood glucose [mM s]	3593.8 ± 632.2	3619.5 ± 10.6	3586.5 ± 809.8	0.527
AUC cardiac output/work [L kJ^−1^]	1.4 ± 0.3	1.67 ± 0.18	1.35 ± 0.32	0.182
AUC heart rate/work [bpm kW^−1^]	1099.3 ± 176.8	1552.2 ± 68.9	969.9 ± 207.6	0.796
AUC systolic BP/work [mmHg kW^−1^]	1341.2 ± 276.9	1862.2 ± 555.5	1192.3 ± 197.3	0.491
AUC diastolic BP/work [mmHg kW^−1^]	675.6 ± 193.4	1017.4 ± 406.5	577.9 ± 132.5	0.931
AUC blood glucose/work [mM kW^−1^]	48.2 ± 12.9	80.5 ± 26.53	38.96 ± 8.98	0.187

**Table 3:** Perfusion-related muscle metabolism of the subjects before training. Mean ± SE of values over all (*n* = 9), carriers (*n* = 7) and non-carriers (*n* = 2) of the *ACE* I-allele and *p*-value of the effect of the I-allele (repeated-measures ANOVA for the training and *ACE*-I-allele factors) on characteristics of performance during ramp exercise.

	*All*	*Non-Carriers*	*I-Allele Carriers*	*p-Value*
AUC tHB VAS [mg mL^−1^ s]	45.4 ± 37.9	34.13 ± 10.93	48.56 ± 45.57	0.945
AUC tHB GAS [mg mL^−1^ s]	55.8 ± 36.9	26.08 ± 15.35	64.25 ± 43.05	0.649
AUC SmO_2_ deficit VAS [%SmO_2_ s]	5786.1 ± 3920.9	3414.5 ± 1611.5	6463.7 ± 4580.7	0.259
AUC SmO_2_ deficit GAS [%SmO_2_ s]	5019.3 ± 2058.8	1904 ± 2126.3	5909.4 ± 2039.5	0.102
AUC blood lactate [mM s]	1887.2 ± 550.2	1567.5 ± 786.2	1978.5 ± 482.8	0.939
AUC tHB/work VAS [mg mL^−1^ kW^−1^]	0.7 ± 0.7	0.72 ± 0.0	0.65 ± 0.85	0.941
AUC tHB/work GAS [mg mL^−1^ kW^−1^]	0.7 ± 0.6	0.52 ± 0.15	0.77 ± 0.73	0.305
AUC SmO_2_ deficit/work VAS [%SmO_2_ kW^−1^]	65.7 ± 38.9	81.76 ± 60.63	61.09 ± 32.75	0.662
AUC SmO_2_ deficit/work GAS [%SmO_2_ kW^−1^]	60.6 ± 27.8	50.05 ± 61.1	63.57 ± 18.34	0.142
AUC blood lactate/work [mM kW^−1^]	23.9 ± 6.1	31.99 ± 6.09	21.58 ± 6.1	0.887

**Table 4:** *ACE*-I/D genotype associated training effects on perfusion-dependent muscle metabolism and cardiac function at rest. Mean ± SE of values over all (*n* = 9), carriers (*n* = 7) and non-carriers (*n* = 2) of the *ACE* I-allele. The *p*-value tested a potential difference between I-allele carrier and non-carrier (repeated-measures ANOVA for the training and *ACE*-allele factors) for metabolic parameters of muscle and cardiac function at rest. Bold underlined values indicate those effects being considered relevant at a threshold of 5%.

	*All*	*Non-Carriers*	*I-Allele Carriers*	*p-Value*
cardiac output [L min^−1^]	4.7 ± 0.4	7.3 ± 0.5	4.0 ± 0.4	**0.013**
heart rate [beat min^−1^]	78.3 ± 2.7	95.0 ± 0.0	73.6 ± 3.5	**0.016**
systolic blood pressure [mmHg]	118.8 ± 7.0	125.0 ± 18.0	117.0 ± 3.8	0.482
diastolic blood pressure [mmHg]	75.4 ± 5.3	74.0 ± 8.0	75.9 ± 4.6	0.853
glucose [mM]	5.3 ± 0.3	6.1 ± 0.5	5.1 ± 0.3	0.132
lactate [mM]	0.8 ± 0.1	0.7 ± 0.1	0.9 ± 0.1	0.299
SmO_2_ at rest VAS [%]	57.2 ± 11.0	53.0 ± 2.8	58.4 ± 13.3	0.349
SmO_2_ at rest GAS [%]	52.0 ± 5.6	43.5 ± 2.1	54.4 ± 6.7	**0.009**
tHB at rest VAS [mg mL^−1^ s]	12.7 ± 0.4	12.7 ± 0.4	12.8 ± 0.4	0.887
tHB at rest GAS [mg mL^−1^ s]	12.6 ± 0.3	12.5 ± 0.1	12.7 ± 0.4	0.241

## Data Availability

Data are available on special request for qualified persons.

## Supplementary Materials

The following supporting information can be downloaded at: https://www.mdpi.com/article/10.3390/genes14051100/s1, Figure S1: Cardiac function before and after interval training between ACE-I/D genotypes. Bar graph of the mean ± SD of normalized values before and after 8-weeks of interval-type training for parameters characterizing cardiac function during incremental ramp exercise. Note the consistent differences in values for cardiac parameters between carriers and non-carriers of the ACE I-allele, which tendency was inversed when values were related to work. +, *p* < 0.10; *, ** *p* < 0.05 and 0.01. Figure S2: Post exercise recovery of cardiac function before and after interval training between ACE-I/D genotypes. Bar graph of the mean ± SD of normalized values before and after 8-weeks of interval-type training for parameters of cardiac function during recovery from ramp incremental exercise. Note the anti-dromic genotype- and training-dependence of systolic and diastolic blood pressure whereas glucose and heart rate was only affected by the ACE I-allele. +, *p* < 0.10; *, ** *p* < 0.05 and 0.01. Figure S3: Post exercise recovery of muscle metabolism before and after interval training between ACE-I/D genotypes. Bar graph of the mean ± SD of normalized values before and after 8-weeks of interval-type training for parameters of perfusion-related muscle metabolism during recovery from ramp incremental exercise. +, *p* < 0.10; *, ** *p* < 0.05 and 0.01. Table S1: Mechanical demand of the training stimulus. Mean ± SE of percentage differences in mechanical characteristics of interval exercise between the last and the first exercise session, and the corresponding *p*-values. Values represent (repeated-measures ANOVA). Bold underlined values were deemed to reflect significant differences at a level of 5%. Table S2: Metabolic impact of the training stimulus. Mean ± SE of percentage differences in metabolic characteristics of interval exercise between the last and the first exercise session, and the corresponding *p*-values (repeated-measures ANOVA). Bold underlined values were deemed to reflect significant differences at a level of 5%. Table S3: Work-related metabolic impact of the training stimulus. Mean ± SE of percentage differences in metabolic characteristics of interval exercise, related to the performed work, between the last and the first exercise session, and the corresponding *p*-values. Values represent (repeated-measured ANOVA). Bold underlined values were deemed to reflect significant differences at a level of 5%. Table S4: ACE-I/D genotype associated training effects on muscle performance. *p*-values of main effects of training × I-allele (repeated ANOVA) and post hoc effects for anaerobic and aerobic performance. Bold underlined values were considered significant. Table S5: ACE-I/D genotype associated training effects on cardiovascular functioning and perfusion-related muscle metabolism during ramp exercise. *p*-values of main effects of training × I-allele (repeated ANOVA) and post hoc effects for metabolic parameters during ramp incremental exercise. Bold underlined values were considered significant. Table S6: ACE-I/D genotype associated training effects on cardiovascular functioning and perfusion-related muscle metabolism during interval exercise. *p*-values of main effects of training × I-allele (repeated ANOVA) and post hoc effects for primarily metabolic parameters being assessed during interval-type exercise for skeletal and cardiac muscle. Bold underlined values were considered significant. Table S7: ACE-I/D genotype associated training effects on the recovery of cardiovascular parameters and perfusion-related skeletal muscle metabolism. *p*-values of main effects of training × I-allele (repeated-measures ANOVA) and post hoc effects for skeletal muscle and cardiovascular parameters being assessed during the first 8-minutes of recovery from ramp incremental exercise. Asterisk indicates effects being tested with a one-sided research hypothesis. Bold underlined values were considered significant.
