# Supplementary material for: ACE-I/D Allele Modulates Improvements of Cardiorespiratory Function and Muscle Performance with Interval-Type Exercise
Source: Genes (Basel). 2023 May 17;14(5):1100. doi: 10.3390/genes14051100 (PMC10218657; doi:10.3390/genes14051100)

Figure S1: *Cardiac function* ***before and after interval training between ACE-I/D genotypes***. Bar graph of the mean ± SD of normalized values before and after 8-weeks of interval-type training for parameters characterizing cardiac function during incremental ramp exercise. Note the consistent differences in values for cardiac parameters between carriers and non-carriers of the ACE I-allele, which tendency was inversed when values were related to work. +, $p<0.10$; *, ** $p<0.05$ and 0.01.

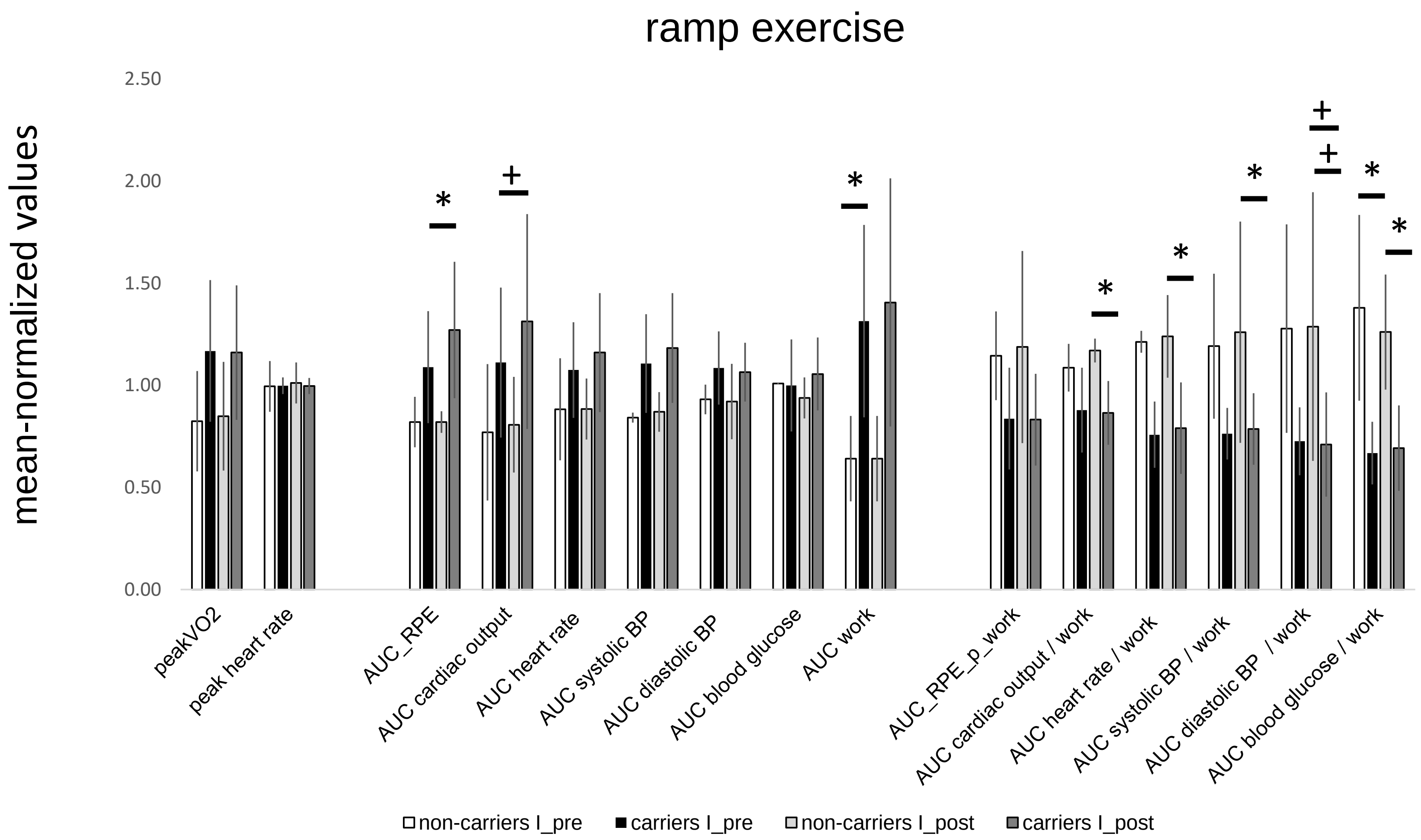

Figure S2: Post exercise recovery of cardiac function before and after interval training *between ACE-I/D genotypes.* Bar graph of the mean ± SD of normalized values before and after 8-weeks of interval-type training for parameters of cardiac function during recovery from ramp incremental exercise. Note the anti-dromic genotype- and training-dependence of systolic and diastolic blood pressure whereas glucose and heart rate was only affected by the ACE I-allele. +, p<0.10; *, ** p<0.05 and 0.01.

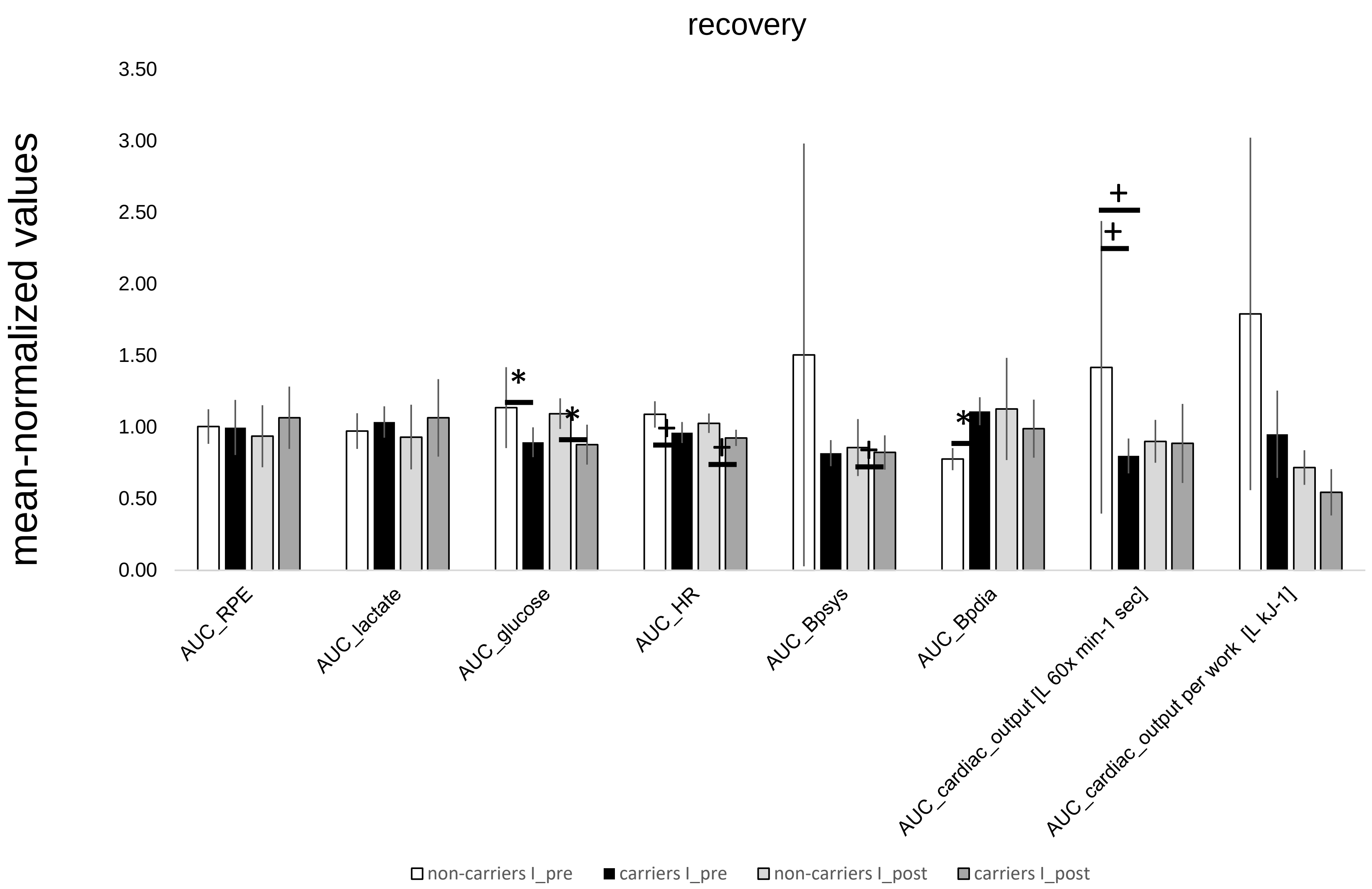

Figure S3: *Post exercise recovery of muscle metabolism before and after interval training between ACE-I/D genotypes.* Bar graph of the mean ± SD of normalized values before and after 8-weeks of interval-type training for parameters of perfusion-related muscle metabolism during recovery from ramp incremental exercise. +, p<0.10; *, ** p<0.05 and 0.01.

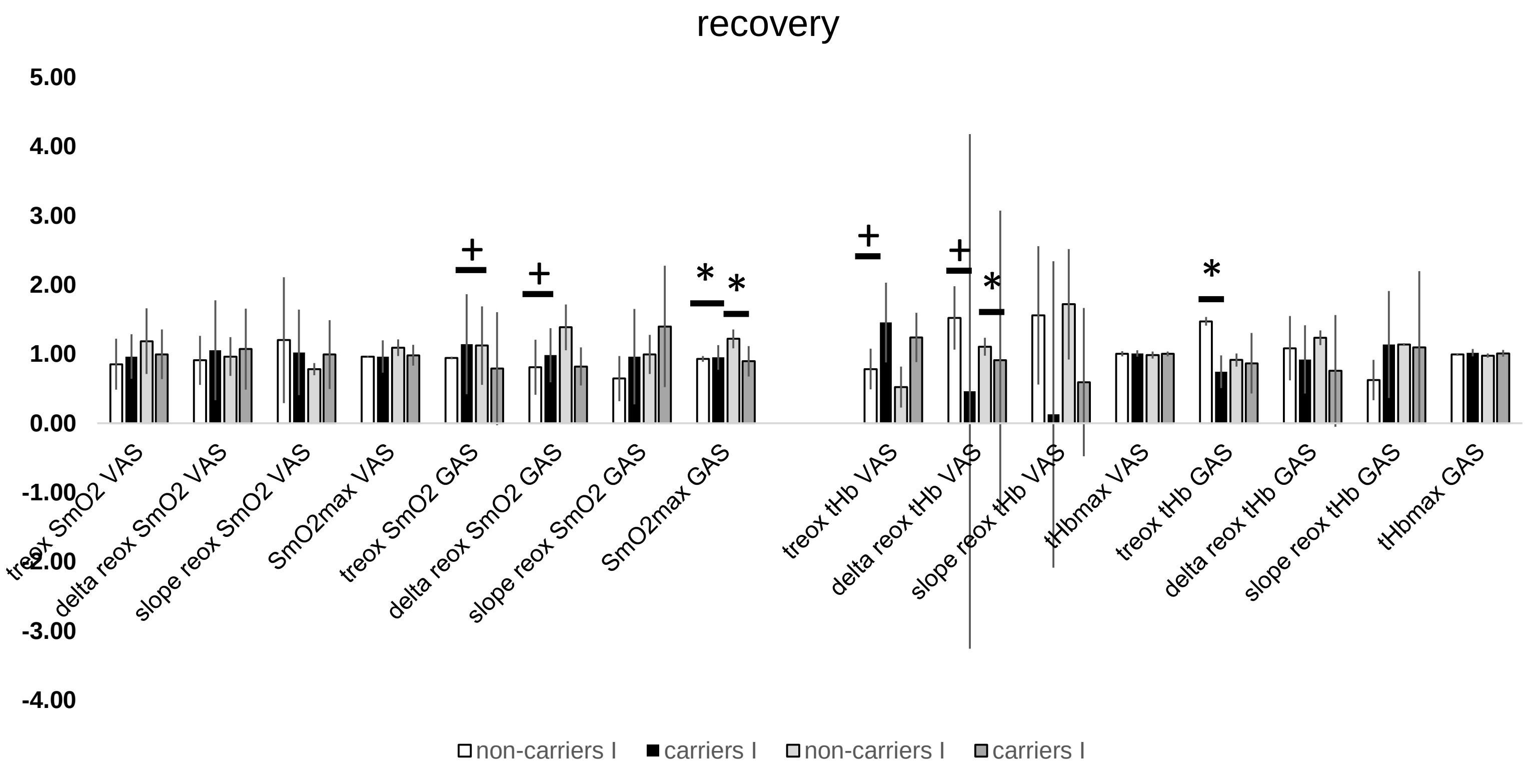

Supplement: Supplementary file 1 [file genes-14-01100-s001.zip › Supplementary Figures.pdf]
