# Supplementary material for: ACE-I/D Allele Modulates Improvements of Cardiorespiratory Function and Muscle Performance with Interval-Type Exercise
Source: Genes (Basel). 2023 May 17;14(5):1100. doi: 10.3390/genes14051100 (PMC10218657; doi:10.3390/genes14051100)
Supplement: Supplementary file 1 [file genes-14-01100-s001.zip › Supplementary Tables.pdf]

Table S1: *Mechanical demand of the training stimulus.* Mean ± SE of percentage differences in mechanical characteristics of interval exercise between the last and the first exercise session, and the corresponding p-values. Values represent (repeated-measures ANOVA). Bold underlined values were deemed to reflect significant differences at a level of 5%.

	Δ post vs pre training [%]	
	mean ± SD	***p-value***
number of sets	**+**56.2 ± 13.0	**<u><0.001</u>**
target power [W]	**+**87.1 ± 15.8	**<u><0.001</u>**
target power/PPO [W x W-1]	**+**87.1 ± 15.7	**<u><0.001</u>**
total work [kJ]	**+**88.5 ± 61.4	**<u><0.001</u>**
exercise duration [sec]	+43.5 ± 27.9	**<u>0.005</u>**
average power [W]	+87.2 ± 58.4	**<u><0.001</u>**
negative work [J]	+66.8 ± 24.9	**<u>0.020</u>**
positive work [J]	+94.6 ± 87.4	**<u>0.038</u>**
% positive work [J]	+0.5 ± 13.0	0.625

Table S2: *Metabolic impact of the training stimulus.* Mean ± SE of percentage differences in metabolic characteristics of interval exercise between the last and the first exercise session, and the corresponding p-values (repeated-measures ANOVA). Bold underlined values were deemed to reflect significant differences at a level of 5%.

	Δ post vs pre training [%]	
	mean ± SD	***p-value***
Cardiovasculature		
AUC cardiac output [L]	+106.2 ± 122.5	**0.020**
AUC heart rate [beats]	+109.0 ± 162.9	**<0.001**
AUC systolic BP [mmHg sec]	+51.9 ± 30.1	**<0.001**
AUC diastolic BP [mmHg sec]	+52.2 ± 36.5	**<0.001**
AUC blood glucose [mM sec]	+52.2 ± 27.7	**<0.001**
skeletal muscle		
AUC SmO2 deficit VAS [% SmO2 sec]	+72.0 ± 83.0	0.104
AUC SmO2 deficit GAS [% SmO2 sec]	+76.7 ± 137.1	0.352
AUC tHb VAS [mg ml-1 sec]	+35.3 ± 26.1	**0.009**
AUC tHb GAS [mg ml-1 sec]	+36.7 ± 32.8	**0.033**
AUC blood lactate [mM sec]	+88.1 ± 203.0	0.511

Table S3: *Work-related metabolic impact of the training stimulus.* Mean ± SE of percentage differences in metabolic characteristics of interval exercise, related to the performed work, between the last and the first exercise session, and the corresponding p-values. Values represent (repeated-measured ANOVA). Bold underlined values were deemed to reflect significant differences at a level of 5%.

cardiac muscle		
AUC RPE / work [W-1]	-14.4 ± 16.9	0.140
AUC cardiac output per work [L kJ-1]	12.6 ± 71.6	0.947
AUC heart rate/work [beats kJ-1]	16.6 ± 99.2	0.818
AUC systolic BP/work [mmHg W-1]	-15.1 ± 20.3	0.098
AUC diastolic BP/work [mmHg W-1]	-13.7 ± 26.7	0.242
AUC glucose/work [mM W-1]	-13.7 ± 22.7	0.304
skeletal muscle		
AUC SmO2 deficit/work VAS [% SmO2 W-1]	-12.5 ± 22.2	**0.049**
AUC SmO2 deficit/work GAS [% SmO2 W-1]	-15.2 ± 45.1	**0.032**
AUC tHb/work VAS [mg ml-1 W-1]	-26.1 ± 19.9	0.147
AUC tHb/work GAS [mg ml-1 W-1]	-26.3 ± 21.6	0.129
AUC lactate / work [mM W-1]	-12.6 ± 62.9	0.074

Table S4: *ACE-I/D genotype associated training effects on muscle performance.* p-values of main effects of training x I-allele (repeated ANOVA) and post hoc effects for anaerobic and aerobic performance. Bold underlined values were considered significant.

	training	***I-allele***	***training x I-allele***	***I vs no I*** *pre*	***I vs no I*** *post*	***post v pre*** *no I-allele*	***post v pre*** *I-allele*
positive peak P	**<u>0.011</u>**	0.429	0.475	0.508	0.358	0.173	0.003
positive peak RFD	0.079	0.611	0.625	0.710	0.515	0.410	0.030
negative peak P	**<u>0.011</u>**	0.739	0.168	0.764	0.110	**<u>0.026</u>**	0.083
negative peak RFD	0.625	0.278	0.670	0.349	0.294	0.971	0.344
reactive peak P	0.583	0.347	0.180	0.518	0.224	0.620	0.065
reactive peak RFD	0.499	0.287	0.083	0.628	0.081	0.482	**<u>0.023</u>**
PPO	0.189	0.127	0.387	0.129	0.128	0.772	**<u>0.040</u>**
body mass	0.907	0.340	0.313	0.327	0.355	0.515	0.339

Table S5: *ACE-I/D genotype associated training effects on cardiovascular functioning and perfusion-related muscle metabolism during ramp exercise.* p-values of main effects of training x I-allele (repeated ANOVA) and post hoc effects for metabolic parameters during ramp incremental exercise. Bold underlined values were considered significant.

	training	***I-allele***	***training x I-allele***	***I vs no I*** *pre*	***I vs no I*** *post*	***post v pre*** *no I-allele*	***post v pre*** *I-allele*
VO2max	0.647	0.250	0.445	0.239	0.265	0.489	0.736
PPO	0.189	0.127	0.387	0.129	0.128	0.772	**<u>0.040</u>**
peak heart rate	0.283	0.903	0.202	0.942	0.732	0.189	0.803
peak cardiac output	0.334	0.131	0.541	0.315	0.062	0.830	0.118
cardiovascular							
AUC work	0.352	0.132	0.352	0.140	0.140	1.000	0.072
AUC RPE	0.120	0.151	0.120	0.237	0.113	1.000	**<u>0.007</u>**
AUC cardiac output	0.315	0.240	0.240*	0.278	0.122*	0.427*	0.094
AUC heart rate	0.422	0.276	0.444	0.340	0.250	0.982	0.121
AUC systolic BP	0.376	0.159	0.680	0.184	0.165	0.779	0.188
AUC diastolic BP	0.764	0.244	0.925	0.294	0.271	0.907	0.677
AUC glucose	0.913	0.713	0.324	0.958	0.419	0.527	0.348
AUC RPE per work	0.768	0.140	0.707	0.160	0.149	0.704	0.930
AUC cardiac output per work	0.463	0.093	0.379	0.253	**<u>0.022</u>***	0.182*	0.867
AUC heart rate per work	0.701	**<u>0.048</u>**	0.411	0.113	**<u>0.025</u>**	0.796	0.218

AUC systolic BP per work	0.921	0.078	0.209	0.149	**<u>0.050</u>**	0.491	0.159
AUC diastolic BP per work	0.405	0.106	0.331	0.181	0.068	0.931	0.080
AUC glucose per work	0.096	**<u>0.017</u>**	0.541	**<u>0.021</u>**	**<u>0.016</u>**	0.189	0.217
skeletal muscle							
AUC tHb VAS	0.832	0.631	0.927	0.684	0.774	0.945	0.747
AUC tHb GAS	0.510	0.326	0.889	0.276	0.460	0.649	0.578
AUC tHb per work VAS	0.727	0.955	0.827	0.885	0.854	0.941	0.552
AUC tHb per work GAS	0.411	0.965	0.316	0.658	0.752	0.305	0.834
AUC SmO2 deficit VAS	0.547	0.592	0.085*	0.404	0.908	0.259	0.186*
AUC SmO2 deficit GAS	0.223	0.127	**<u>0.044</u>***	**<u>0.045</u>**	0.411	0.102	0.258*
AUC SmO2 def per work VAS	0.381	0.193	**<u>0.034</u>***	0.662	**<u>0.049</u>**	0.123	0.119*
AUC SmO2 def per work GAS	0.425	0.778	**<u>0.039</u>***	0.512	0.162	0.142	0.118*
AUC lactate	0.631	0.215	0.540	0.371	0.207	0.939	0.264
AUC lactate per work	0.879	0.182	0.923	0.141	0.244	0.887	0.953

Table S6: *ACE-I/D genotype associated training effects on cardiovascular functioning and perfusion-related muscle metabolism during interval exercise.* p-values of main effects of training x I-allele (repeated ANOVA) and post hoc effects for primarily metabolic parameters being assessed during interval-type exercise for skeletal and cardiac muscle. Bold underlined values were considered significant.

	training	***I-allele***	***training x I-allele***	***I vs no I*** *pre*	***I vs no I*** *post*	***post v pre*** *no I-allele*	***post v pre*** *I-allele*
cardiovascular							
AUC cardiac output	**<u>0.019</u>**	0.832	0.992	0.839	0.860	0.130	**<u>0.015</u>**
AUC RPE per work	0.140	0.540	0.837	0.742	0.223	0.437	0.087
AUC cardiac output per work	0.947	0.845	0.430	0.948	0.563	0.676	0.369
AUC systolic BP per work	0.098	0.588	0.675	0.603	0.634	0.225	0.162
AUC diastolic BP per work	0.282	0.840	0.671	0.956	0.342	0.695	0.132
AUC heart rate per work	0.818	0.964	0.072	0.610	0.288	0.223	0.087
AUC glucose per work	0.304	0.391	0.629	0.747	**<u>0.026</u>**	0.742	0.131
AUC glucose	**<u><0.001</u>**	0.428	0.939	0.498	0.475	**<u>0.007</u>**	**<u><0.001</u>**
skeletal muscle							
AUC tHb GAS	**<u>0.033</u>**	0.808	0.788	0.911	0.783	0.222	**<u>0.017</u>**
AUC tHb VAS	**<u>0.009</u>**	0.950	0.686	0.866	0.859	0.056	**<u>0.012</u>**
AUC SmO2 deficit GAS	0.352	0.931	0.328	0.378	0.729	0.976	0.066
AUC SmO2 deficit VAS	0.104	0.913	0.519	0.637	0.890	0.522	**<u>0.031</u>**
AUC lactate	0.511	0.513	0.419	0.191	0.959	0.928	0.144

AUC tHb per work GAS	0.129	0.707	0.548	0.994	0.301	0.541	0.054
AUC tHb per work VAS	0.147	0.571	0.338	0.994	0.149	0.731	**0.035**
AUC SmO2 deficit per work GAS	**0.032***	0.186*	**0.019***	**0.041***	0.417*	**0.030**	0.360*
AUC SmO2 deficit per work VAS	**0.049***	0.093*	0.097*	**0.039***	0.195*	0.096	0.320*
AUC lactate per work	0.074	0.169	0.103	**0.040**	0.621	0.059	0.816

Table S7: *ACE-I/D genotype associated training effects on the recovery of cardiovascular parameters and perfusion-related skeletal muscle metabolism.* p-values of main effects of training x I-allele (repeated-measures ANOVA) and post hoc effects for skeletal muscle and cardiovascular parameters being assessed during the first 8-minutes of recovery from ramp incremental exercise. Asterisk indicates effects being tested with a one-sided research hypothesis. Bold underlined values were considered significant.

	training	***I-allele***	***training x I-allele***	***I vs no I*** *pre*	***I vs no I*** *post*	***post v pre*** *no I-allele*	***post v pre*** *I-allele*
cardiovascular							
AUC RPE	1.000	0.701	0.252	0.962	0.484	0.502	0.227
AUC cardiac output	0.222	0.194	0.102	0.097	0.946	0.111	0.584
AUC heart rate	0.150	**0.037**	0.697	0.075	0.063	0.289	0.239
AUC systolic BP	0.250	0.124	0.244	0.174	0.758	0.195	0.987
AUC diastolic BP	0.297	0.354	0.053	**0.003**	0.481	0.091	0.243
AUC glucose	0.478	**0.033***	0.750	**0.037***	**0.044***	0.560	0.671

skeletal muscle									
AUC lactate	0.944	0.502	0.683		0.497	0.546		0.786	0.719
delta tHb GAS	0.993	0.578	0.612		0.885	0.535		0.912	0.820
delta tHb VAS	0.991	0.551	0.600		0.607	0.888		0.868	0.737
treox tHb GAS	0.368	**0.038**	0.130		**0.026**	0.870		0.199	0.592
treox tHb VAS	0.399	**0.003**	0.054		0.070	**0.050**		0.598	0.417
slope tHb GAS	0.714	0.703	0.612		0.613	0.955		0.651	0.943
slope tHb VAS	0.709	0.129	0.224		0.290	0.259		0.913	0.559
overshoot tHb GAS	0.220	0.410	0.144		0.476	0.378		0.425	0.256
overshoot tHb VAS	0.217	0.739	0.291		0.857	0.635		0.332	0.401
delta SmO2 GAS	0.200	0.657	0.084		0.738	0.182		0.052	0.282
delta SmO2 VAS	0.793	0.733	0.642		0.741	0.753		0.822	0.892
treox SmO2 GAS	0.644	0.869	0.173		0.623	0.489		0.585	0.060
treox SmO2 VAS	0.499	0.945	0.563		0.849	0.781		0.485	0.900
slope SmO2 GAS	0.170	0.517	0.167		0.624	0.499		0.480	0.110
slope SmO2 VAS	0.286	0.966	0.374		0.710	0.632		0.259	0.879
overshoot SmO2 GAS	0.074	0.322	0.085		0.901	**0.050**		**0.016**	0.331
overshoot SmO2 VAS	0.186	0.685	0.333		0.985	0.420		0.184	0.742
